# Mediators of the Effects of Gender on Uric Acid Nephrolithiasis: A Novel Application of Structural Equation Modeling

**DOI:** 10.1038/s41598-018-24485-x

**Published:** 2018-04-17

**Authors:** Hao-Wei Chen, Yu-Chen Chen, Frances M. Yang, Wen-Jeng Wu, Ching-Chia Li, Yong-Yuan Chang, Yii-Her Chou

**Affiliations:** 1 Graduate Institute of Clinical Medicine, College of Medicine, Kaohsiung Medical University, Kaohsiung, Taiwan; 2 Department of Urology, Kaohsiung Medical University Hospital, Kaohsiung Medical University, Kaohsiung, Taiwan; 3 Department of Population Health Sciences, Division of Epidemiology, Medical College of Georgia, Augusta University, Augusta, USA; 4 Department of Healthcare Administration & Medical Informatics, College of Health Sciences, Kaohsiung Medical University, Kaohsiung, Taiwan

## Abstract

Numerous epidemiological studies have shown that male patients with uric acid nephrolithiasis outnumber female patients. To our knowledge, no research exists evaluating the reasons gender affects the development of uric acid nephrolithiasis. We hereby used a novel application of structural equation modeling to analyze the mediators of the effects of gender on uric acid nephrolithiasis. In 1,098 patients with nephrolithiasis between 2012 and 2016, male gender was found to have a statistically significant positive indirect effect on the development of uric acid nephrolithiasis, which was mediated by lower urine pH (estimate: 0.010, standard error: 0.005, critical ratio: 2.135, 95% confidence interval: 0.002–0.023, *P* = 0.017), lower estimated glomerular filtration rate (estimate: 0.014, standard error: 0.005, critical ratio: 2.993, 95% confidence interval: 0.006–0.025, *P* < 0.001), and higher incidence rate of gout (estimate: 0.009, standard error: 0.005, critical ratio: 2.028, 95% confidence interval: 0.002–0.021, *P* = 0.009). We conclude that low urine pH, impaired renal function, and gout are the mediators of the effect of male gender on the development of uric acid nephrolithiasis. The survey, treatment, and follow-up of kidney diseases, acidic urine, and uric acid metabolism disorders should be considered in men with uric acid nephrolithiasis.

## Introduction

Previous large scale epidemiology studies in diverse populations showed that uric acid stones mostly occur in men. The earliest reports showed a male-to-female ratio of 3:1 in uric acid nephrolithiasis^1^. In a recent Australian epidemiology study, the number of male patients was higher than that of female patients with uric acid nephrolithiasis^2^. However, no studies have investigated the reasons for the gender-related difference in the development of uric acid nephrolithiasis.

Although understanding the effect of gender on the development of uric acid nephrolithiasis would be beneficial for elucidating the pathoetiology of uric acid nephrolithiasis, surveys, treatment, and recurrence prevention, it’s complicated and challenging for several reasons: (1) Several different factors can affect its development^3–9^. (2) The cascade of different factors that have indirect and direct effects on uric acid stone formation. For examples, diabetes mellitus (DM) indirectly forms uric acid nephrolithiasis by lowering the urine pH^4,10^. (3) Factors that form uric acid nephrolithiasis are also interrelated^11,12^. Such a complicated problem is limited by using traditional statistical methods for analysis, which have been consistently published in previous studies. Several nonmedical journals have recently used structural equation modeling (SEM) as a statistical method to analyze the relationship between different factors. Using SEM is advantageous because it can establish a best-fitting model to inform theory and measurement; simultaneously examine different mediating and confounding factors; adjust measurement errors; and measure interactions between variables^13^.

We hereby used SEM to test our hypothesis that there are different clinical, social, and behavioral factors that mediate the relationship between male and female patients with nephrolithiasis and their development of uric acid nephrolithiasis. Our model includes testing whether gender directly or indirectly affects the development of uric acid nephrolithiasis through differences in underlying diseases, different diet styles, and different occupations.

## Methods

### Selection and Description of Participants

Between January 2012 and December 2016, patients diagnosed with nephrolithiasis who have undergone genitourinary surgery including ureteroscopic lithotripsy, percutaneous nephrolithotomy, and open nephrolithotomy at the Kaohsiung Medical University Hospital, Kaohsiung, Taiwan, were eligible for enrollment in this study. Exclusion criteria were patients with genitourinary tract tumors, kidney transplant, genitourinary tract anomaly, recurrent nephrolithiasis, mixed stones containing more than one stone components, or renal replacement therapy, such as hemodialysis. Patients younger than 18 years old and those without detailed medical records were also excluded. Results of stone analysis using infrared spectroscopy were reported by the medical technologist and confirmed by two urologists. This study, including any relevant details, was approved by the Kaohsiung Medical University Hospital Institutional Review Board (KMUHIRB-E(II)-20170212). We confirmed that all experiments were performed in accordance with relevant guidelines and regulations. Informed consent was obtained from all participants and/or their legal guardians.

Information about sociodemographic characteristics (age and gender), health (body mass index (BMI) and comorbidities, including DM, hypertension (HT), and gout), and clinical information (urinalysis data, including bacteriuria, urine pH, and urine specific gravity (SG)) were collected before nephrolithiasis treatment. Stone symptoms were divided into “symptomatic” and “asymptomatic”. Of the urology practice in our hospital, there were few of patients referred by internists received surgery for asymptomatic nephrolithiasis due to nephrolithiasis found accidentally in abdominal computed tomography (CT), plain film, or abdominal ultrasound scanning when treating other diseases. In this study, asymptomatic patients were defined as those with nephrolithiasis diagnosed accidently during a health checkup or in the follow-up for other diseases, and those who did not show specific symptoms of nephrolithiasis, such as abdominal pain, flank pain, and hematuria. Estimated glomerular filtration rate (eGFR) was calculated using the isotope dilution mass spectrometry traceable Modification of Diet in Renal Disease (IDMS-MDRD) formula (eGFR [mL/min/1.73 m^2^] = 175 × (Scr)^−1.154^ × (Age)^−0.203^ × [0.742 if female])^14^. The conditions of the patients’ occupations were recorded on the basis of whether the patients worked in heat environment^8^ or in a local environment with a temperature of >28 °C without air-conditioning for >5 hours per working day. Alcohol consumption was decided on whether the alcohol intake of the patients was >14 units a week according to the UK Chief Medical Officers’ (CMO) low-risk unit guidelines^15^. Our patients were all from Kaohsiung, Taiwan. In this area, the diet styles could be differentiated by Western diet, including high-animal protein and high-fatty foods,^16^ such as fried chicken and fries, compared to traditional diet of pork, chicken, seafood, rice, and soy^17^. The patients were asked whether they ate more than 12 Western diet style meals per week to decide the diet type.

### Statistical Analyses and Outcome

We used the IBM SPSS version 22 statistical software (IBM Corporation, NY, USA) for descriptive statistics and data compilation. For descriptive statistics, the Fisher’s exact test was used for comparing the categorical parameters, including gender, stone symptoms, HT, DM, bacteriuria, occupation, diet style, and alcohol consumption. The Mann-Whitney U test was used for continuous variables, including age, BMI, eGFR, urine SG, and urine pH between uric acid nephrolithiasis and non uric acid nephrolithiasis. All *P* values of <0.05 were considered statistically significant.

**Figure 1:**
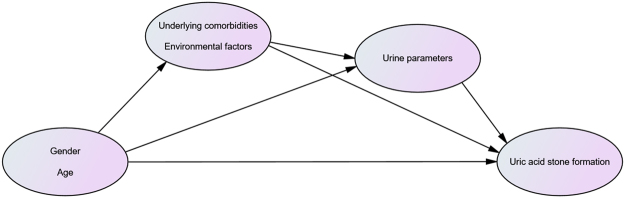
Hypothesized model. Each path will have a starting point and arrowhead direction. The starting point variables represent independent variables (exposure factors). The arrowhead direction represents dependent variables (results).

We used the IBM SPSS Amos version 21 statistical software (IBM Corporation, NY, USA) through the SEM analysis method to analyze proven and possible factors that can affect uric acid nephrolithiasis. Gender and age were included. The underlying factors such as DM, HT, gout, stone symptoms, bacteriuria, BMI, eGFR, and environmental factors such as occupation, diet style, and alcohol consumption were analyzed. The urine parameters included urine pH and urine SG. We drew a hypothetical model (Fig. 1) based on the proven paths that affect uric acid nephrolithiasis (e.g., underlying factors that will impact urine parameters and indirectly impact the uric acid nephrolithiasis path) and our hypothetical paths. Each path will have a starting point and arrowhead direction. The starting point variables represent independent variables (exposure factors). The arrowhead direction represents dependent variables (results).

SEM analysis includes three steps as follows: (1) We used the maximum likelihood method in IBM SPSS Amos to test our hypothetical model. The best-fitting model was defined as the minimum chi-square to degree of freedom ratio (CMIN/DF): 1–3, *P* > 0.05, goodness of fit index (GFI) > 0.8, adjusted goodness of fit index (aGFI) >0.8, and root mean square error of approximation (RMSEA) <0.08^13^. (2) If our hypothetical model was not the best-fitting, we used modification indices to select, increase/decrease, and adjust the path and correlations until the model conforms to the best-fitting model. (3) Lastly, we used bias-corrected bootstrapping (5000 bootstrap samples) to test whether all possible direct effects, correlations, and specific indirect effects of each parameter reached statistical significance. The standardized estimate, standard error (SE), critical ratio (CR), 95% confidence interval (CI) of each path were calculated^13^.

### Data Availability

The datasets generated during and/or analyzed during the current study are available from the corresponding author on reasonable request.

## Results

Of the 1098 patients who were included, 146 and 952 had pure uric acid and non-uric acid nephrolithiasis, respectively. By comparing these two patient groups, statistically significant differences in age, gender, eGFR, HT, DM, and gout were found. Besides, significantly lower proportion of bacteriuria (8.9% vs 17%, *P* = 0.01) and urine pH (5.51 vs 6.09, *P* < 0.001) were found in the patients with uric acid nephrolithiasis (Table 1). In the patients with uric acid nephrolithiasis, male patients were significant younger and had lower proportion of DM (Table 2). Of 952 patients with non-uric acid stone, we found significant difference of age, gout, diet style, alcohol consumption, occupation, bacteriuria, urine SG, and urine PH between the both genders (Table 3).

**Table 1:** Characteristics of 1098 patients with nephrolithiasis based on uric acid nephrolithiasis.

Characteristic	Uric acid nephrolithiasis (n = 146)	Non-uric acid nephrolithiasis (n = 952)	*P* value
Age, mean (SD), year	60.4 (12.6)	52.8 (12.7)	<0.001
Gender, No. (%)			0.046
Male	110 (75)	638 (67)	
BMI, mean (SD), kg/m^2^	25.6 (3.8)	25.4 (3.5)	0.27
eGFR, mean (SD), ml/min/1.73 m^2^	55.1 (29.4)	80.1 (29.4)	<0.001
Stone symptoms, No. (%)			0.22
Asymptomatic	9 (6.2)	90 (9.5)	
Hypertension, No. (%)	59 (40)	236 (25)	<0.001
Diabetes mellitus, No. (%)	42 (29)	122 (13)	<0.001
Gout, No. (%)	14 (9.6)	21 (2.2)	<0.001
Alcohol Consumption, No. (%)	8 (5.5)	98 (10)	0.071
Occupation, No. (%)			0.42
Working in heat environment	21 (14)	113 (12)	
Diet style, No. (%)			0.9
Western diet style	32 (22)	213 (22)	
Bacteriuria, No. (%)	13 (8.9)	164 (17)	0.011
Urine SG, mean (SD), g/dl^3^	1.014 (0.006)	1.014 (0.007)	0.9
Urine pH, mean (SD)	5.51 (0.54)	6.09 (0.77)	<0.001

Abbreviations: SD, standard deviation; BMI, body mass index; eGFR, estimated glomerular filtration rate; SG, specific gravity.

**Table 2:** Characteristics of 146 patients with uric acid nephrolithiasis based on genders.

Characteristic	Male (n = 110)	Female (n = 36)	*P* value
Age, mean (SD), year	58.7 (12.6)	65.9 (10.7)	0.001
BMI, mean (SD), kg/m^2^	25.9 (3.6)	24.5 (4.2)	0.12
eGFR, mean (SD), ml/min/1.73 m^2^	56.2 (30.2)	51.9 (27.6)	0.48
Stone symptoms, No. (%)			1
Asymptomatic	7 (6.4)	2 (5.6)	
Hypertension, No. (%)	33 (30)	26 (72)	<0.001
Diabetes mellitus, No. (%)	22 (20)	20 (56)	<0.001
Gout, No. (%)	12 (11)	2 (5.6)	0.52
Alcohol Consumption, No. (%)	8 (7.3)	0 (0)	0.2
Occupation, No. (%)			0.002
Working in heat environment	21 (19)	0 (0)	
Diet style, No. (%)			0.001.
Western diet style	31 (28)	1 (2.8)	
Bacteriuria, No. (%)	8 (7.3)	5 (14)	0.31
Urine SG, mean (SD), g/dl^3^	1.014 (0.006)	1.014 (0.006)	0.8
Urine pH, mean (SD)	5.49 (0.55)	5.58 (0.49)	0.19

Abbreviations: SD, standard deviation; BMI, body mass index; eGFR, estimated glomerular filtration rate; SG, specific gravity.

**Table 3:** Characteristics of 952 patients with non-uric acid nephrolithiasis based on genders.

Characteristic	Male (n = 638)	Female (n = 314)	*P* value
Age, mean (SD), year	51.1 (12.4)	56.1 (12.8)	<0.001
BMI, mean (SD), kg/m^2^	25.4 (3.4)	25.4 (3.9)	0.78
eGFR, mean (SD), ml/min/1.73 m^2^	80.8 (28.4)	78.8 (31.3)	0.17
Stone symptoms, No. (%)			0.9
Asymptomatic	61 (9.6)	29 (9.2)	
Hypertension, No. (%)	147 (23)	89 (28)	0.08
Diabetes mellitus, No. (%)	77 (12)	45 (14)	0.35
Gout, No. (%)	20 (3.1)	1 (0.3)	0.004
Alcohol Consumption, No. (%)	94 (15)	4 (1.3)	<0.001
Occupation, No. (%)			<0.001
Working in heat environment	105 (16)	8 (2.5)	
Diet style, No. (%)			<0.001
Western diet style	174 (27)	39 (12)	
Bacteriuria, No. (%)	74 (12)	90 (29)	<0.001
Urine SG, mean (SD), g/dl^3^	1.015 (0.006)	1.013 (0.006)	<0.001
Urine pH, mean (SD)	6.04 (0.74)	6.19 (0.82)	0.014

Abbreviations: SD, standard deviation; BMI, body mass index; eGFR, estimated glomerular filtration rate; SG, specific gravity.

**Figure 2:**
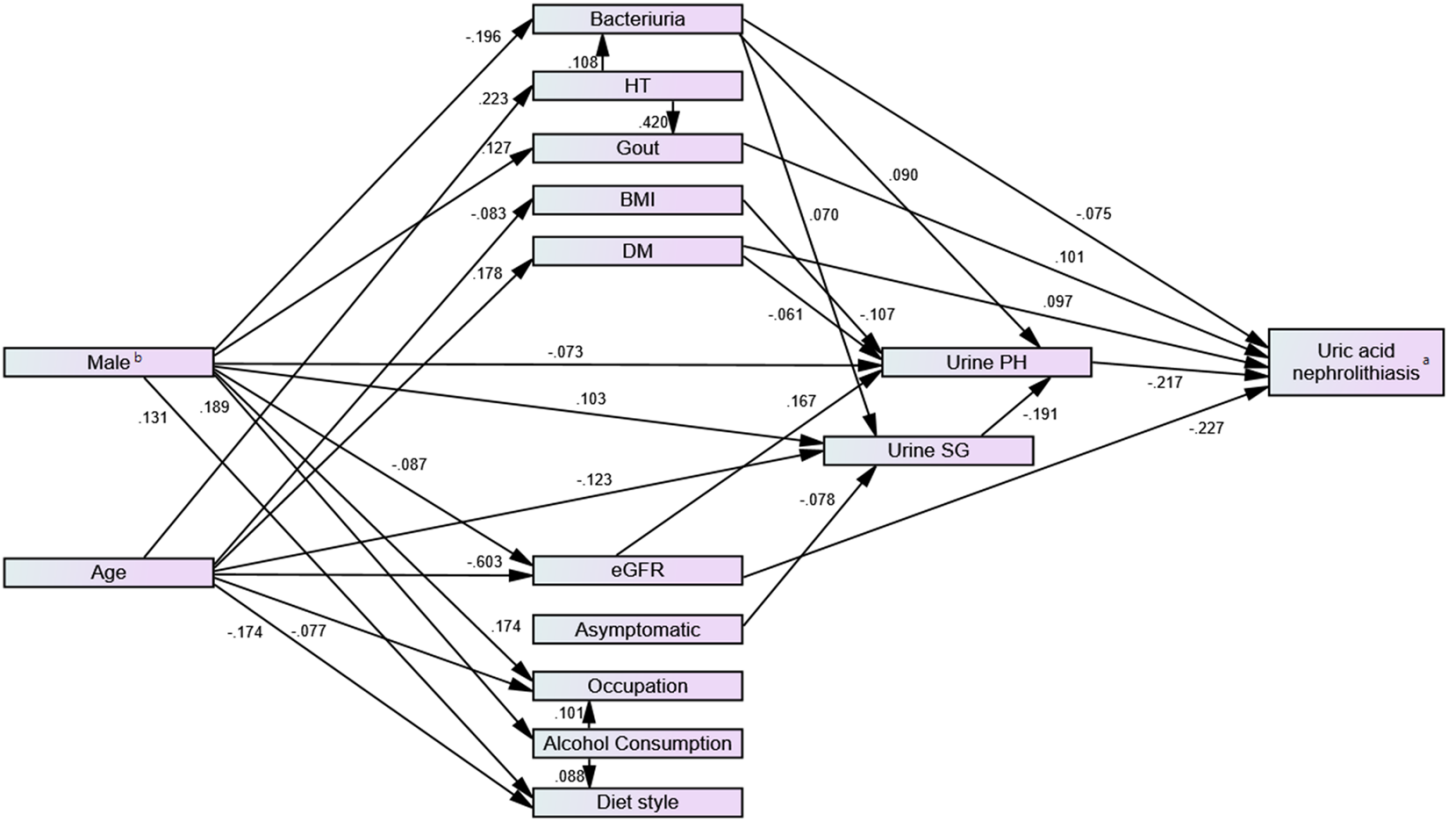
Best-fitting model with statiscally significant pathways and standarized estimates. ^*^Abbreviations: HT, hypertension; DM, diabetes mellitus; BMI, body mass index; eGFR, estimated glomerular filtration rate; SG, specific gravity. ^*^Correlations and measure errors of each parameter are hidden in the figure to avoid chaotic representation. ^a^Uric acid nephrolithiasis versus non-uric acid nephrolithiasis. ^b^Male gender versus female gender.

Using SEM, the final best-fitting model was constructed for uric acid nephrolithiasis (minimum χ^2^ (CMIN):47.089, degrees of freedom: 38, CMIN/DF: 1.239, *P* = 0.148, GFI: 0.994, aGFI: 0.982, and RMSEA: 0.015). Our results did not demonstrate that gender had a direct effect on uric acid nephrolithiasis formation. However, we found that male gender had a strong negative direct effect (estimate: −0.119) on urine pH, eGFR, and bacteriuria. Male gender also had a strong positive direct effect on gout. Besides, urine pH (estimate: −0.217) had a significantly negative direct effect on the development of uric acid nephrolithiasis. In addition to urine pH, we also found that gout (estimate: 0.101) and DM (estimate: 0.097) were significant risk factors with a positive direct effect on the development of uric acid nephrolithiasis. Bacteriuria (estimate: −0.075) and eGFR (estimate: −0.227) had a significant negative direct effect on the development of uric acid nephrolithiasis. All statistically significant standardized pathways were reported in Fig. 2. Several other statistically significant direct effects and correlations were found between the parameters in this study. These results are shown in Tables 4 and 5.

**Table 4:** Statistically significant direct effects between the parameters.

Direct effects	Estimate^c^	SE	CR	95% CI	*P*
Urine pH → Uric acid nephrolithiasis^a^	−0.217	0.024	−9.065	−0.263 ~ −0.169	<0.001
Gout → Uric acid nephrolithiasis^a^	0.101	0.043	2.375	0.018 ~ 0.183	0.016
Bacteriuria → Uric acid nephrolithiasis^a^	−0.075	0.022	−3.369	−0.117 ~ −0.030	0.003
DM → Uric acid nephrolithiasis^a^	0.097	0.035	2.753	0.030 ~ 0.167	0.001
eGFR → Uric acid nephrolithiasis^a^	−0.227	0.028	−8.225	−0.280 ~ −0.173	<0.001
Male^b^ → Urine pH	−0.073	0.032	−2.299	−0.135 ~ −0.011	0.018
Male^b^ → Bacteriuria	−0.196	0.032	−6.206	−0.260 ~ −0.134	<0.001
Male^b^ → Gout	0.127	0.031	4.038	0.077 ~ 0.205	<0.001
Male^b^ → eGFR	−0.087	0.026	−3.391	−0.138 ~ −0.038	<0.001
Male^b^ → Urine SG	0.103	0.033	3.151	0.036 ~ 0.166	0.003
Male^b^ → Alcohol Consumption	0.189	0.031	10.782	0.152 ~ 0.221	<0.001
Male^b^ → Occupation	0.174	0.020	8.570	0.132 ~ 0.211	<0.001
Male^b^ → Diet style	0.131	0.026	5.014	0.077 ~ 0.181	<0.001
Age → HT	0.223	0.027	8.387	0.172 ~ 0.274	<0.001
Age → DM	0.178	0.028	6.361	0.122 ~ 0.232	<0.001
Age → BMI	−0.083	0.031	−2.626	−0.144 ~ −0.021	0.009
Age → eGFR	−0.603	0.021	−29.111	−0.643 ~ −0.561	<0.001
Age → Diet style	−0.174	0.029	−5.970	−0.229 ~ −0.114	0.001
Age → Occupation	−0.077	0.029	−2.692	−0.133 ~ −0.020	0.009
Urine SG → Urine pH	−0.191	0.030	−6.386	−0.249 ~ −0.131	<0.001
BMI → Urine pH	−0.107	0.030	−3.575	−0.165 ~ −0.048	0.001
DM → Urine pH	−0.061	0.028	−2.155	−0.117 ~ −0.007	0.03
eGFR → Urine pH	0.167	0.030	5.578	0.107 ~ 0.223	0.001
Bacteriuria → Urine pH	0.090	0.031	2.947	0.029 ~ 0.150	0.005
Bacteriuria → Urine SG	0.070	0.030	2.316	0.011 ~ 0.131	0.018
HT → Bacteriuria	0.108	0.032	3.367	0.045 ~ 0.173	0.001
Asymptomatic → Urine SG	−0.078	0.026	−2.998	−0.128 ~ −0.024	0.003
Alcohol Consumption → Diet style	0.088	0.034	2.565	0.021 ~ 0.158	0.008
Alcohol Consumption → Occupation	0.101	0.040	2.506	0.025 ~ 0.184	0.009

Abbreviations: SE, standard error; CR, critical ratio; CI, confidence interval; P, two tailed P value; BMI, body mass index; eGFR, estimated glomerular filtration rate; SG, specific gravity; DM, diabetes mellitus; HT, hypertension.

^a^Uric acid nephrolithiasis versus non-uric acid nephrolithiasis.

^b^Male gender versus female gender.

^c^Standardized estimate.

**Table 5:** Statistically significant correlations between the parameters.

Correlations		Estimate^b^	SE	CR	95% CI	*P*
Age	Male^a^	−0.173	0.029	−5.983	−0.229 ~ −0.116	<0.001
Bacteriuria	Asymptomatic	0.155	0.039	3.941	0.078 ~ 0.232	<0.001
BMI	Diet style	0.056	0.023	2.402	0.011 ~ 0.101	0.015
BMI	HT	0.144	0.031	4.875	0.087 ~ 0.203	<0.001
BMI	DM	0.110	0.029	3.789	0.056 ~ 0.168	<0.001
BMI	eGFR	0.263	0.032	8.160	0.197 ~ 0.322	<0.001
DM	Alcohol Consumption	0.105	0.034	3.007	0.041 ~ 0.178	0.002
DM	Asymptomatic	0.120	0.038	3.188	0.047 ~ 0.194	0.001
DM	HT	0.357	0.032	10.666	0.293 ~ 0.422	<0.001
Diet style	Asymptomatic	0.082	0.035	2.387	0.016 ~ 0.149	0.014
Diet style	Occupation	0.608	0.028	21.922	0.552 ~ 0.661	<0.001
HT	Asymptomatic	0.114	0.034	3.349	0.046 ~ 0.180	<0.001
HT	Alcohol Consumption	0.085	0.033	2.635	0.021 ~ 0.148	0.013

Abbreviations: SE, standard error; CR, critical ratio; CI, confidence interval; P, two tailed P value; BMI, body mass index; eGFR, estimated glomerular filtration rate; SG, specific gravity; DM, diabetes mellitus; HT, hypertension.

^a^Male gender versus female gender.

^b^Standardized estimate.

Several statistically significantly specific indirect effects were found between gender, age, BMI, DM, eGFR, and uric acid nephrolithiasis formation. When testing the pathway from gender to uric acid nephrolithiasis formation, which was mediated by urine pH factor (gender → urine pH → uric acid nephrolithiasis), we found that male gender had a statistically significantly positive indirect effect on uric acid stone formation (estimate: 0.011, SE: 0.005, CR: 2.135, 95% CI: 0.002–0.023, *P* = 0.017). Besides, male gender also had a significantly positive indirect effect on uric acid nephrolithiasis, which was mediated by differences in eGFR (estimate: 0.014, SE: 0.005, CR: 2.993, 95% CI: 0.006–0.025, *P* < 0.001), rate of gout (estimate: 0.009, SE: 0.005, CR: 2.028, 95% CI: 0.002–0.021, *P* = 0.009), and rate of bacteriuria (estimate: 0.011, SE: 0.004, CR: 2.891, 95% CI: 0.004–0.019, *P* = 0.002) between men and women. The standardized estimates, SE, CR, 95% CI, and *P* value of all significantly specific indirect pathways are presented in Table 6.

**Table 6:** Statistically significant specific indirect effects between the parameters.

Specific indirect effects	Estimate^c^	SE	CR	95% CI	*P*
Male^b^ → Bacteriuria → Uric acid nephrolithiasis^a^	0.011	0.004	2.891	0.004 ~ 0.019	0.002
Male^b^ → Gout → Uric acid nephrolithiasis^a^	0.009	0.005	2.028	0.002 ~ 0.021	0.009
Male^b^ → Urine pH → Uric acid nephrolithiasis^a^	0.011	0.005	2.135	0.002 ~ 0.023	0.017
Male^b^ → eGFR → Uric acid nephrolithiasis^a^	0.014	0.005	2.993	0.006 ~ 0.025	<0.001
Male^b^ → Bacteriuria → Urine pH → Uric acid nephrolithiasis^a^	0.003	0.001	2.452	0.001 ~ 0.006	0.004
Male^b^ → eGFR → Urine pH → Uric acid nephrolithiasis^a^	0.002	0.001	2.668	0.001 ~ 0.004	<0.001
Age → eGFR → Uric acid nephrolithiasis^a^	0.004	0.001	7.218	0.003 ~ 0.005	<0.001
BMI → Urine pH → Uric acid nephrolithiasis^a^	0.002	0.001	3.145	0.001 ~ 0.004	0.001
DM → Urine pH → Uric acid nephrolithiasis^a^	0.013	0.006	2.056	0.002 ~ 0.026	0.026

Abbreviations: SE, standard error; CR, critical ratio; CI, confidence interval; P, two tailed P value; BMI, body mass index; eGFR, estimated glomerular filtration rate; SG, specific gravity; DM, diabetes mellitus; HT, hypertension.

^a^Uric acid nephrolithiasis versus non-uric acid nephrolithiasis.

^b^Male gender versus female gender.

^c^Standardized estimate.

## Discussion

The causes of nephrolithiasis are complicated, and several factors can affect its formation. In nearly 50 years of accumulated evidence from the literature, researchers have used epidemiological studies to collect accurate data on the analysis of factors that can affect nephrolithiasis^1–4,7,8,10,18,19^. Several factors (e.g., DM, obesity, and low urine pH) have been proven to affect the development of uric acid nephrolithiasis^3,4,10^. Unfortunately, all studies only used traditional regression analysis, which was unable to examine the complex mediating and confounding factors. Although numerous epidemiological studies have shown that male patients with uric acid nephrolithiasis outnumber female patients^2,20^, to the best of our knowledge, there is no research evaluating the reasons gender affect the development of uric acid nephrolithiasis.

Actually, traditional statistical limits have caused many studies on uric acid nephrolithiasis to avoid investigating gender^7^, or have experimental and control groups that were gender matched^9^. In addition, in fact, difference indeed existed between both genders. In our study, when testing the gender difference in our control group patients with non-uric acid stones, underlying diseases, urine parameters, and life style were very different between the both genders. And, these could cause some bias when investigating the gender effect on the development of uric acid nephrolithiasis using simple traditional statistical methods.

SEM can be used to simultaneously examine multiple observational variables, mediating variables, and confounding factors. In addition, it can also be used to study the correlation between each variable, adjust the measurement errors between each variable, and produce the best-fitting theory model^13^. Thus, we believe that SEM is the best statistical method for complex, multivariable, large-sample uric acid nephrolithiasis studies. We hereby used SEM to draw the best-fitting uric acid nephrolithiasis theory model and obtained the following findings: (1) gender had an indirect effect on the development of uric acid nephrolithiasis owing to differences in urine pH, eGFR, gout, and bacteriuria between men and women; (2) many complicated correlations, mediators, and confounders exist between each parameters for the development of uric acid nephrolithiasis.

It is interesting that the males had lower urine pH and lower eGFR than the females and were the mediators of the effect of gender on the development of uric acid nephrolithiasis after adjusting the dietary factors, occupational factors, and underlying comorbidities using the SEM methods. We believe that the same pathoetiology, which is the disturbance of uric acid metabolism, explains the differences in urine pH, renal function, and gout between the genders. Male gender has been clearly proven to be a risk factor of gout. Most studies report that female hormones (estrogen) increases the efficiency of the kidneys when eliminating uric acid and maintains a lower uric acid concentration in the blood. Thus, uric acid is less likely to accumulate in the joints. Consequently, gout occurs less frequently in women^21^. In reality, uric acid accumulate not only in the joints, but also around the renal tubule. This could cause damage of renal function and interfere ammonium secretion by the renal tubule. Finally, dysfunction of ammonium secretion lowers the urine pH in the kidney and indirectly induces the development of uric acid nephrolithiasis^22^.

We also found that the lower rate of bacteriuria was the mediator of the effect of male gender on the development of uric acid nephrolithiasis. Women have a shorter urethra, thus younger women have a much higher probability of urinary tract infections and acute pyelonephritis than men of the same age^23^. These urinary tract infections are sometimes caused by bacteria that produce urease, and the infection alkalinizes the urine pH^24^. The uric acid in urine can dissolve in urine in an alkaline environment, and will not produce crystals. Therefore, it is less likely for women to develop uric acid nephrolithiasis.

Understanding the risk factors of uric acid nephrolithiasis and how they mutually affect each other can be beneficial for elucidating the pathoetiology of uric acid nephrolithiasis, stone surveys, treatment, and prevention of recurrence. This study determines how gender affects uric acid stones through urine pH, eGFR, gout, and bacteriuria. When male patients had nephrolithiasis, and stone analysis shows uric acid, we recommended that survey, treatment, and follow-up of kidney diseases, urine pH, and uric acid metabolism disorders should be considered. Checkups can result in early diagnosis of these diseases, and treatment of these diseases can also prevent the recurrence of uric acid nephrolithiasis. Future studies on uric acid nephrolithiasis should establish an animal experiment model and molecular biomedical research for different hormones in men and women, differences in urinary tract structure, and genetic differences. This can clarify the development of uric acid nephrolithiasis from biochemical molecules and help treat and prevent recurrence of uric acid nephrolithiasis.

Our study limitations include: (1) All our patients had nephrolithiasis. We lacked patients without nephrolithiasis for comparison. First, finding a patient without nephrolithiasis requires the use of abdominal CT and ureteroscopy to ascertain that nephrolithiasis and Randall’s plug are absent^25^. The members of institutional review board believes that this type of invasive check and radiation exposure used to survey the control group violates medical ethics. Clinically, we treated a group of patients with nephrolithiasis, but did not select patients with uric acid nephrolithiasis from the general population. Different components of nephrolithiasis require different treatments and recurrence prevention. Therefore, separating patients with uric acid nephrolithiasis from a large group of patients with nephrolithiasis and basing on a theory model for clinical application are reasonable. (2) We did not have urine and blood chemical substance data such as sodium, potassium, and uric acid for all the patients. The first reason for this is such checks would significantly increase the medical costs. The second reason is based on the European Association of Urology (EAU) guidelines that not all patients with nephrolithiasis must undergo metabolic analysis^26^. We believe that certain chemicals in urine can affect the development of nephrolithiasis, but previous studies proved that low urine pH was a main cause of the development of uric acid nephrolithiasis^4^. Thus, we believe that within the SEM theory model, the lack of urine chemical variable may not influence the results for the mediators of the effect of gender on the development of uric acid nephrolithiasis. (3) A homogenous sample was used for this study, which has limited generalizability. However, although the ethnicity is different than that of Western nations, previous articles indicate that the difference in the distribution of nephrolithiasis cases between ethnicities is caused by differences in diet type and occupation^27^. Our SEM method included these environmental factors. Therefore, we believe that our results are also applicable to patients in Western countries. (4) We did not collect the smoking data of the patients. Although smoking is a lifestyle factor that could impact many diseases, there is limited evidence in the literature suggesting that smoking could impact the development of uric acid nephrolithiasis. Therefore, we did not collect the data of smoking in this study. However, smoking often co-occurs with alcohol consumption and to avoid statistical multi-collinearity, we only included alcohol consumption in this study.

## Conclusions

Impaired renal function, low urine pH, and gout are the mediators of the effects of the male gender on the development of uric acid nephrolithiasis. These findings suggest that differences between male and female patients in their development of uric acid nephrolithiasis as an outcome is due to clinical factors that can potentially help explain the underlying mechanism in the pathway. In addition, these clinical factors need to be considered in surveys for data collection, treatment decisions, follow-up of evaluation of kidney diseases, acidic urine, and uric acid metabolism disorders, especially in men with uric acid nephrolithiasis.
